# Single Donor Infusion of S-Nitroso-Human-Serum-Albumin Attenuates Cardiac Isograft Fibrosis and Preserves Myocardial Micro-RNA-126-3p in a Murine Heterotopic Heart Transplant Model

**DOI:** 10.3389/ti.2022.10057

**Published:** 2022-04-13

**Authors:** Anne-Kristin Schaefer, Attila Kiss, André Oszwald, Felix Nagel, Eylem Acar, Arezu Aliabadi-Zuckermann, Matthias Hackl, Andreas Zuckermann, Renate Kain, Andrzej Jakubowski, Peter Ferdinandy, Seth Hallström, Bruno K. Podesser

**Affiliations:** ^1^ Ludwig Boltzmann Institute for Cardiovascular Research, Center for Biomedical Research, Medical University of Vienna, Vienna, Austria; ^2^ Department of Cardiac Surgery, Medical University of Vienna, Vienna, Austria; ^3^ Department of Pathology, Medical University of Vienna, Vienna, Austria; ^4^ Comprehensive Cancer Center, Medical University of Vienna, Vienna, Austria; ^5^ TamiRNA GmbH, Vienna, Austria; ^6^ Department of Pharmacology, Jagiellonian University Medical College, Kraków, Poland; ^7^ Department of Anesthesiology and Intensive Care, Małopolska Orthopedic and Rehabilitation Hospital, Kraków, Poland; ^8^ Department of Pharmacology and Pharmacotherapy, Faculty of Medicine, Semmelweis University, Budapest, Hungary; ^9^ Division of Physiological Chemistry, Otto Loewi Research Center, Medical University of Graz, Graz, Austria

**Keywords:** heart transplantation, graft preservation, cardiac isograft injury, cardiac graft fibrosis, experimental transplantation

## Abstract

**Objectives:** Cold ischemia and subsequent reperfusion injury are non-immunologic cornerstones in the development of graft injury after heart transplantation. The nitric oxide donor S-nitroso-human-serum-albumin (S-NO-HSA) is known to attenuate myocardial ischemia-reperfusion (I/R)-injury. We assessed whether donor preservation with S-NO-HSA affects isograft injury and myocardial expression of GATA2 as well as miR-126-3p, which are considered protective against vascular and endothelial injury.

**Methods:** Donor C57BL/6 mice received intravenous (0.1 μmol/kg/h) S-NO-HSA (*n* = 12), or 0.9% saline (control, *n* = 11) for 20 min. Donor hearts were stored in cold histidine-tryptophan-*α*-ketoglutarate-N solution for 12 h and underwent heterotopic, isogenic transplantation, except 5 hearts of each group, which were analysed immediately after preservation. Fibrosis was quantified and expression of GATA2 and miR-126-3p assessed by RT-qPCR after 60 days or immediately after preservation.

**Results:** Fibrosis was significantly reduced in the S-NO-HSA group (6.47% ± 1.76 vs. 11.52% ± 2.16; *p* = 0.0023; 12 h-S-NO-HSA-hHTX vs. 12 h-control-hHTX). Expression of miR-126-3p was downregulated in all hearts after ischemia compared to native myocardium, but the effect was significantly attenuated when donors received S-NO-HSA (1 ± 0.27 vs. 0.33 ± 0.31; *p* = 0.0187; 12 h-S-NO-HSA-hHTX vs. 12 h-control-hHTX; normalized expression to U6 snRNA).

**Conclusion:** Donor pre-treatment with S-NO-HSA lead to reduced fibrosis and preservation of myocardial miR-126-3p and GATA2 levels in murine cardiac isografts 60 days after transplantation.

## Graphical Abstract

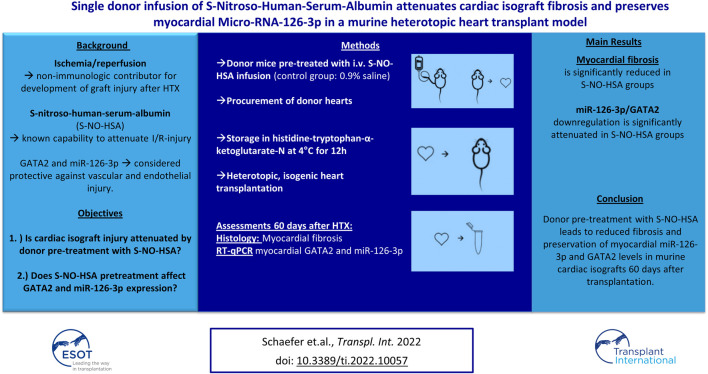

## Introduction

Chronic allograft injury (CAI), consisting of vasculopathy and interstitial fibrosis, affects approximately 50% of patients after 10 years and limits long-term survival following heart transplantation (1). There is substantial evidence that endothelial injury during organ procurement and preservation, caused by ischemia and subsequent reperfusion, results in endothelial dysfunction. The latter is a non-immunologic contributor to pathogenesis and progression of CAI (2–4). Besides endothelial dysfunction, the progression of interstitial and perivascular fibrosis consecutively leads to impaired diastolic and systolic graft function, thus preservation of endothelial and vascular function is certainly a clinically desirable goal.

Recent studies have demonstrated that supplementation of nitric oxide (NO), or increased expression of endothelial NO-synthase (eNOS) protects against both IR-injury and fibrosis (5, 6). We have proposed the concept of donor- and recipient management using the NO-donor S-nitroso-human-serum-albumin (S-NO-HSA) (7), a high-molecular-weight S-nitrosothiol with a high S-nitrosograde and exact equimolar nitrosation (8).

NO release by S-NO-HSA can downregulate eNOS activity by feedback inhibition (9), and thereby prevent eNOS uncoupling and subsequent superoxide and peroxynitrite formation caused by eNOS uncoupling during I/R. Supporting evidence for this concept comes from previous small-and large animal preclinical studies, where addition of S-NO-HSA to the preservation solution has shown to enhance hemodynamic and metabolic recovery after cardioplegic arrest in the isolated rabbit heart after 6 h of hypothermic, cardioplegic arrest (5), and intravenous infusion of S-NO-HSA at a dose of 0.1 μmol/kg/h reduced ischemia/reperfusion injury in the pig heart after unprotected warm ischemia (7, 10). Whether S-NO-HSA provides similar protective effects beyond acute functional and metabolic improvements in the setting of heart transplantation (HTX) has not yet been investigated.

Endothelial cell function and eNOS expression in different organs, including the heart, is highly regulated on epigenetic levels, particularly by the GATA2 transcription factor (11). In general, GATA2 also activates the expression of miR-126, the most abundant microRNA in endothelial cells (12). Recent clinical studies demonstrated the diagnostic relevance of miR-126 in association with the presence of CAI in HTX recipients (13, 14). Nevertheless, little is known about 1) the spatial-temporal expression of both GATA2 and miR-126 in transplanted hearts; 2) the effect of S-NO-HSA on their expression levels.

The aim of the present study was to investigate whether donor pretreatment with S-NO-HSA attenuates long-term development of graft fibrosis, and to characterize the expression of GATA2 and miR-126-3p with and without S-NO-HSA pretreatment in a mouse model of isogenic, heterotopic HTX after prolonged cold ischemia and reperfusion.

## Materials and Methods

### Experimental Animals

Male C57BL/6 mice aged 8–9 weeks (Department for Laboratory Animal Science and Genetics, Himberg, Austria) were used in this study. The experimental protocol was approved by the regional Ethics Committee for Laboratory Animal Experiments at the Medical University of Vienna and the Federal Ministry Republic of Austria, Education, Science and Research (authorization protocol number GZ 66.009/0158-WF/V/3b/2015) and conforms with the “Principles of Laboratory Animal Care” formulated by the National Society for Medical Research and the Guide for the Care and Use of Laboratory Animals published by the US National Institutes of Health (NIH Publication No. 85-23, revised 1996).

### S-NO-HSA Preparation

HSA was processed as previously described (10, 15). S-NO-HSA preparation is depicted in detail in Supplementary Appendix S1. S-NO-HSA was dissolved in 0.9% saline solution and continuously infused *via* a catheter in the femoral vein for 20 min (0.1 µmol S-NO-HSA/kg/h) prior to donor heart procurement.

### Experimental Groups

**FIGURE 1:**
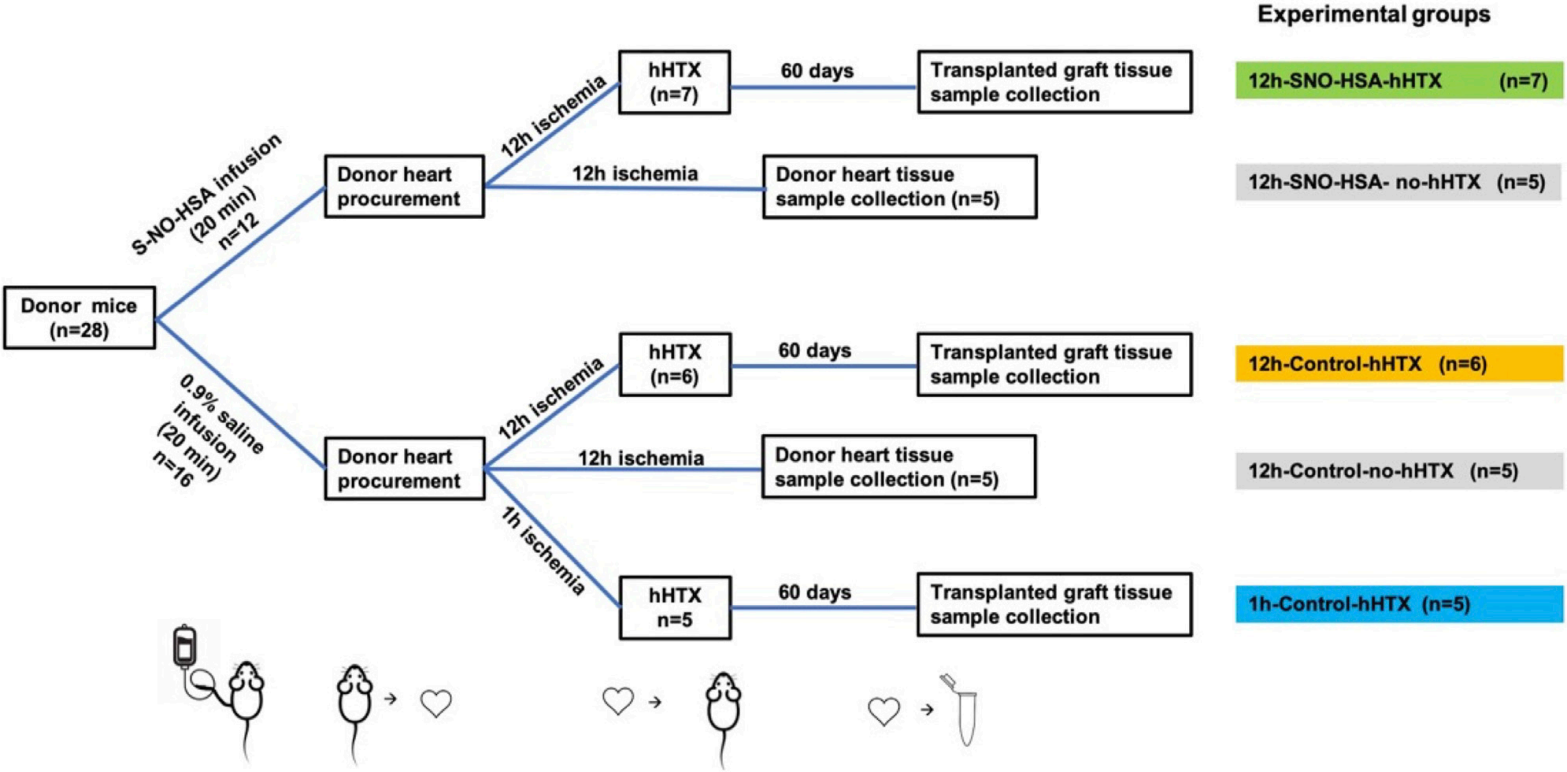
Experimental setup. Palpation score, myocardial fibrosis, GATA2 and miR-126-3p were assessed in 3 groups 60 days after heterotopic transplantation (hHTX). HHTX took place either after 12 h of cold ischemia with donor preparation using SNO-HSA (12 h-SNO-HSA-hHTX), donor preparation with normal saline (12 h-control-hHTX), or after 1 h of cold ischemia (1 h-control-hHTX). Additionally, GATA2 and miR-126-3p expression was assessed in 3 non-transplanted groups directly after the cold ischemia period after 12 h with donor preparation using SNO-HSA (12 h-SNO-HSA-no-hHTX) or normal saline (12 h-control-no-hHTX). GATA2 and miR-126-3p expression was analyzed in some grafts without cold ischemia (no ischemia) as an additional control group. An overview of experimental groups is depicted in Table 1.

In order to clarify the impact of S-NO-HSA on graft preservation and miR-126-3p and GATA2 expression, the experimental setup depicted in Figure 1 was used.

Hearts without ischemia (*n* = 19; no ischemia) served as additional controls for the expression analysis of GATA2 and miR-126-3p assessed by RT-qPCR. The experimental groups are summarized in Table 1.

**TABLE 1:** Overview of experimental groups.

Experimental group	Total (n)	Histology (n)	miRNA analysis (n)
Transplanted groups			
12 h-SNO-HSA-hHTX	7	7	5
12 h-control-hHTX	6	6	6
1 h-control-hHTX	5	5	5
Non-transplanted groups			
12 h-SNO-HSA-no-hHTX	5	—	5
12 h-control-no-hHTX	5	—	5
no-ischemia	19	—	19

### Donor Heart Procurement

Donor mice were anesthetized by intraperitoneal injection of the mixture of xylazine (5 mg/kg) and ketamine (100 mg/kg), followed by catheterization of the femoral vein and intravenous infusion of S-NO-HSA (0.1 μmol/kg/h) dissolved in 0.9% saline solution, or 0.9% saline solution only (control groups) for 20 min, followed by thoracotomy and administration of 1 ml of HTK-N solution (4°C, Dr. Franz Köhler Chemie GmbH, Bensheim, Germany) supplemented with 100 units of heparin *via* the inferior vena cava to arrest the heart. The ascending aorta and pulmonary trunk were divided. After ligation of the superior venae cavae, and en block ligation of the pulmonary veins, the graft was excised, flushed with heparinized HTK-N solution, and stored in HTK-N solution at 4°C for either 1 h or 12 h.

### Heterotopic Abdominal Heart Transplantation

Recipient surgeries were conducted as described previously (16, 17). Analgesia was provided by subcutaneous injection of buprenorphine (0.1 mg/kg bodyweight) and anaesthesia maintained with inhaled isoflurane. Briefly, after laparotomy and dissection of the infrarenal aorta and IVC, the abdominal aorta and IVC were cross-clamped infrarenally and directly proximal to the iliac bifurcation. After longitudinal aortotomy and venotomy, the donor’s acending aorta was anastomosed to the recipient’s abdominal aorta and the donor’s pulmonary trunk to the recipient’s IVC using running 10-0 nylon sutures. The duration of warm ischemia during the implantation process was standardized to 30 min.

### Assessment of Functional Graft Status

Graft viability was assessed and rated by transabdominal palpation using a score from 0 (no palpable contraction) to 5 (strong beat and adequate heart rate) before sample collection 60 days after transplantation as described previously (16).

### Myocardial Tissue Sample Collection

Sixty days after transplantation, recipient mice were anaesthetized with the mixture of ketamine and xylazine (0.1 ml/10 g bodyweight), and anaesthesia was confirmed by hind foot and tail pinch. The transplanted heart was excised and transversally cut at mid-papillary level. The base of the hearts was frozen in liquid nitrogen and stored at −80°C, and the apex fixed in 7.5% formaldehyde for histopathology analysis.

### Assessment of miR-126-3p and GATA2 Expression

Assessment of miR-126-3p and GATA2 expression are described in Supplementary Appendix S1.

### Histological Analysis

Histological analysis is described in detail in Supplementary Appendix S1. For quantification of interstitial fibrosis, sections of 4 µm were cut and stained with Sirius red. The areas of total and positively stained tissue within a region were quantified using CellProfiler (18).

### Human Cardiac Fibroblast Experiments

Human ventricular cardiac fibroblasts (Lonza, Basel, Switzerland) were cultured in fibroblast basal medium supplemented with 0.1% insulin, 0.1% fibroblast growth factor, 0.1% GA-1000, and 10% FBS (all Lonza, Basel, Switzerland) as described previously (19). Cultures were washed once with DPBS (Thermo Fisher Scientific, CA, United States) when indicated, and split at a confluency level of 70%. Cells were treated for 24 h follows: 1) No treatment—control; 2) 20 ng/ml TGF-β (Abcam, Cambridge, United Kingdom); 3) 25 μmol/L HSA; 4) 25 μmol/L S-NO-HSA; 5) 20 ng/ml TGF-β + 25 μmol/L HSA and 6) 20 ng/ml TGF-β + 25 μmol/L S-NO-HSA. Total RNA was extracted, and expression of target genes (Supplementary Table S5) were assessed by RT-qPCR (Supplementary Appendix S1).

### Statistical Analysis

Data are presented as mean ± standard deviation. Testing for normality was performed using the Kolmogorov-Smirnov-test. One-way ANOVA with Tukey HSD post-hoc test was used for multiple comparisons between the groups. Two-tailed *p* < 0.05 was considered statistically significant. Spearman correlation was used to assess correlation of miR-126-3p and GATA2 expression. Analysis was performed using Prism 8 software for macOS (GraphPad Inc., San Diego, CA, United States).

## Results

### Baseline Characteristics and Functional Graft Assessment

Baseline characteristics of experimental animals are shown in Supplementary Table S1.

**FIGURE 2:**
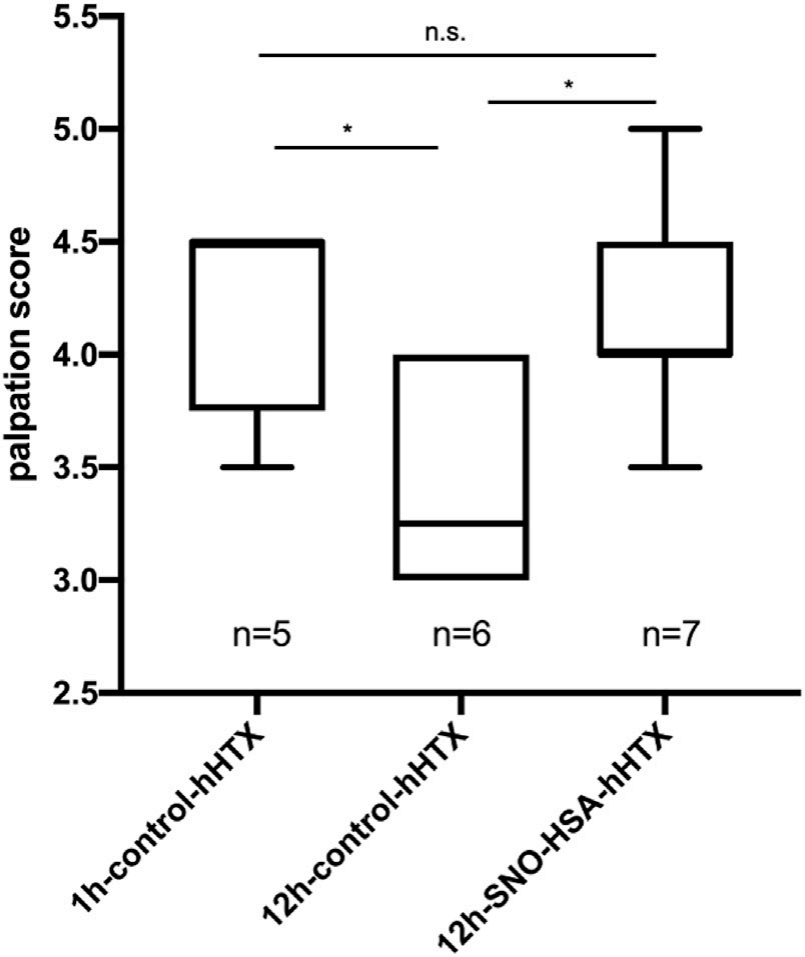
Palpation score of control and S-NO-HSA pretreatment groups. Graft viability was assessed and rated by transabdominal palpation using a score from 0 (no palpable contraction) to 5 (strong beat and adequate heart rate) prior sample collection at day 60 after transplantation (hHTX) in the 3 transplanted groups. *p < 0.05; n.s. = non-significant.

Palpation score 60 days after transplantation was significantly higher in grafts transplanted after 1 h ischemia and 12 h ischemia when donors received S-NO-HSA compared to the 12 h control-group. (4.2 ± 0.45 vs. 3.42 ± 0.49; *p* = 0.041; 1 h-control-hHTX vs. 12 h-control-hHTX and 4.21 ± 0.49 vs. 3.42 ± 0.49; *p* = 0.023 12 h-S-NO-HSA-hHTX vs. 12 h-control-hHTX). Palpation score is depicted in Figure 2.

### Myocardial Interstitial Fibrosis

**FIGURE 3:**
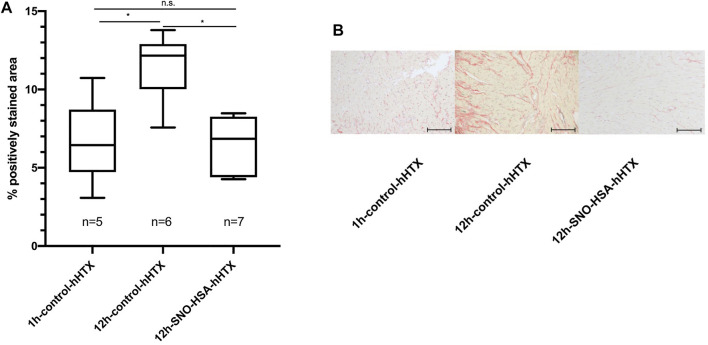
Panel **(A)**: Extent of myocardial interstitial fibrosis 60 days after hHTX. Control (1 h-control-hHTX, 12 h-control-hHTX) and S-NO-HSA pretreated (12 h-SNO-HSA-hHTX) groups. Extent of fibrosis in isografts 60 days after hHTX At least two regions of myocardium each of the interventricular septum (IVS) and the right ventricle (RV) were selected for quantification. The areas of total and positively stained tissue within a region were quantified using CellProfiler (18). *p < 0.05; n.s. = non-significant Panel **(B)**: Representative images of sirius red stain of transplanted myocardium 60 days after hHTX. Sections of 4 µm were cut and stained with Sirius red for quantification of interstitial fibrosis at day 60 after transplantation (hHTX). Images were acquired using an upright microscope using a ×40 objective and a CCD-camera with a ×0.63 adapter (Axio Imager. M2 and Axiocam 512 color, Carl Zeiss, Aalen, Germany). Scale bar = 100 μm.

In hearts transplanted after prolonged cold ischemia (12 h), fibrosis was significantly reduced 60 days after transplantation when donors were pretreated with S-NO-HSA (6.47% ± 1.76 vs. 11.52% ± 2.16; *p* = 0.0023; 12 h-S-NO-HSA-hHTX vs. 12 h-control-hHTX). The extent of myocardial interstitial fibrosis is depicted in Figure 3A, and representative images of each group are shown in Figure 3B. Regarding duration of ischemia, the extent of fibrosis in hearts transplanted after prolonged (12 h) ischemia was significantly higher than the extent of fibrosis in the second control group transplanted after 1 h of ischemia (11.52% ± 2.16 vs. 6.66% ± 2.72; *p* = 0.006; 12 h-control-hHTX vs. 1 h-control-hHTX) at 60 days after transplantation. Fibrosis in donors pretreated with S-NO-HSA and prolonged (12 h) ischemia was not significantly different to the reference group transplanted after 1 h of ischemia at 60 days after transplantation (6.47% ± 1.76 vs. 6.66% ± 2.72; *p* = 0.99; 12 h-S-NO-HSA-hHTX vs. 1 h-control-hHTX).

### MiR-126-3p Expression in the Myocardium

**FIGURE 4:**
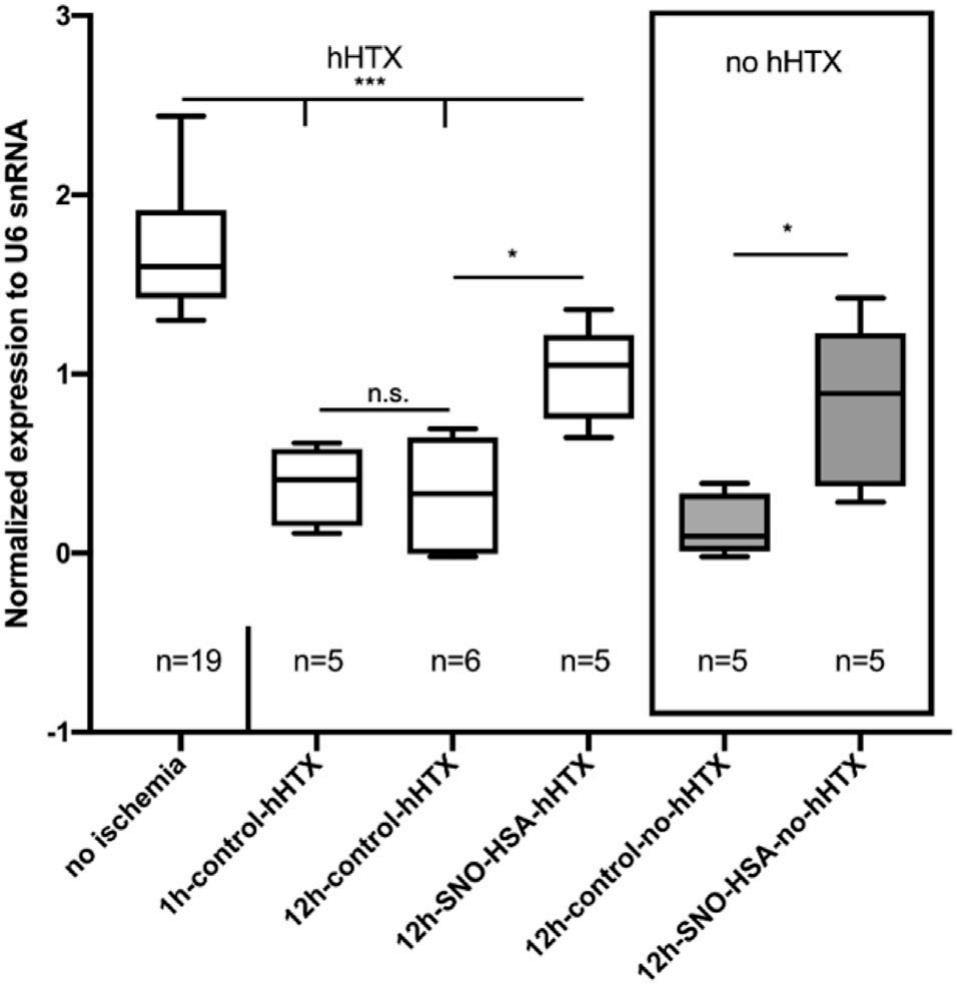
Myocardial miR-126-3p expression levels in control (1 h-control-hHTX, 12 h-control-hHTX, 12 h-control-no-hHTX) and S-NO-HSA pretreated (12 h-SNO-HSA-hHTX, 12 h-SNO-HSA-no-hHTX) groups. Box plots from myocardium not subjected to ischemia (no ischemia), from non-transplanted reference hearts with 12 h ischemia (12 h-control-no-hHTX) and S-NO-HSA pretreated non-transplanted hearts (12 h-SNO-HSA-no-hHTX). ***p < 0.001; *p < 0.05; n.s = .non-significant.

Figure 4 depicts the expression of miR-126-3p in myocardial tissue. When compared to myocardium not subjected to ischemia (no ischemia), miR-126-3p was significantly reduced in all grafts (transplanted and non-transplanted) subjected to ischemia. However, transplanted grafts from donors pretreated with S-NO-HSA showed a significantly increased miR-126-3p expression compared to control groups without S-NO-HSA-pretreatment (transplanted groups: 1 ± 0.27 vs. 0.33 ± 0.31; *p* = 0.0187; 12 h-SNOHSA-hHTX vs. 12 h-control-hHTX; normalized expression to U6 snRNA).

In the groups analyzed directly after the ischemic period (12 h) without subsequent transplantation, expression of miR-126-3p was significantly higher in the group with S-NO-HSA pretreated donors when compared to grafts procured without prior S-NO-HSA-administration. (0.82 ± 0.46 vs. 0.16 ± 0.17; *p* = 0.029; 12 h-SNOHSA-no-hHTX vs. 12 h-control-no-hHTX; normalized expression to U6 snRNA).

There was no significant difference in miR-126-3p expression levels between the control groups transplanted after 12 h vs. only 1 h of ischemia (0.33 ± 0.31 vs. 0.38 ± 0.22; *p* = 0.99; 12 h-control-hHTX vs. 1 h-control-hHTX; normalized expression to U6 snRNA).

### Myocardial GATA2 Expression

**FIGURE 5:**
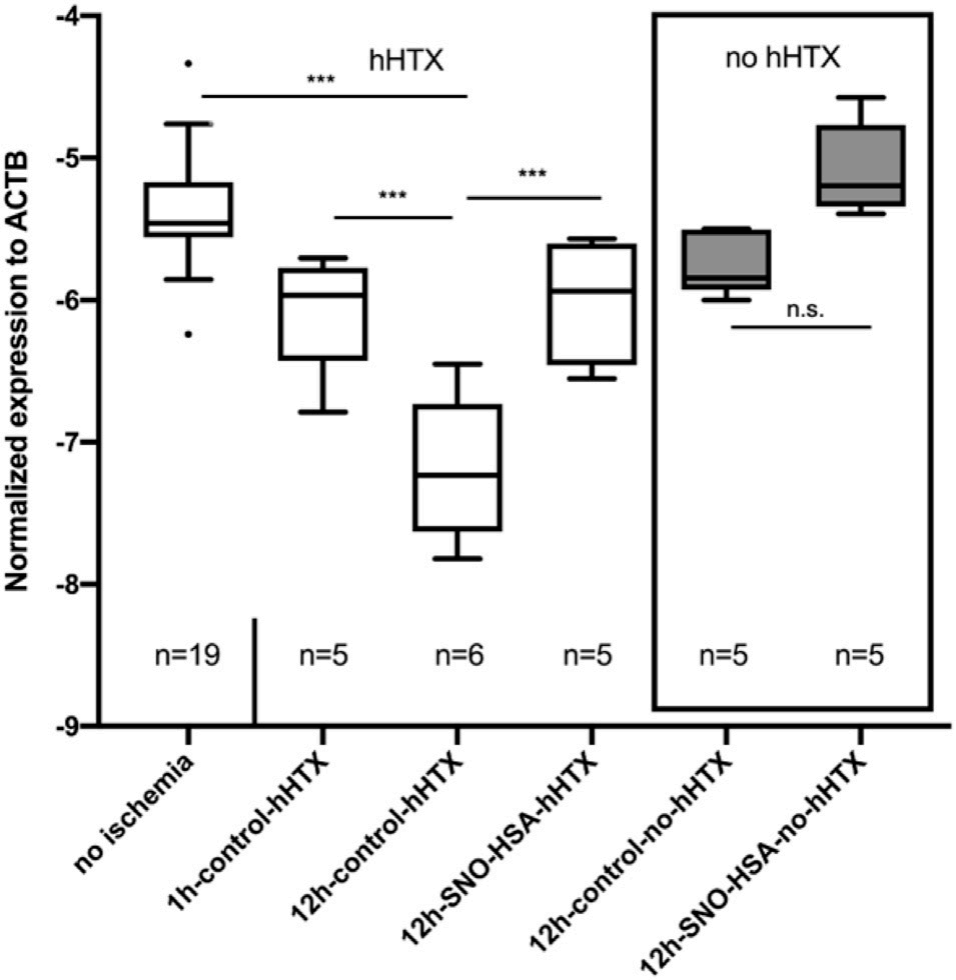
Myocardial GATA2 expression levels in control and S-NO-HSA pretreatment groups. Box plots from myocardium not subjected to ischemia (no ischemia), from non-transplanted reference hearts with 12 h ischemia (12 h-Control-no-hHTX) and S-NO-HSA pretreated non-transplanted hearts (12 h-SNO-HSA-no-hHTX). Assessment of GATA2 expression levels from the myocardial biopsies are described in detail in Supplementary Appendix S1. ***p < 0.001; *p < 0.05; n.s. = non-significant.

Myocardial GATA2 expression is depicted in Figure 5. Sixty days after hHTX, GATA2 expression was significantly downregulated in grafts subjected to 12 h of ischemia, but this effect was reversed when donors had received S-NO-HSA (−6.00 ± 0.45 vs. −7.188 ± 0.5; 12 h-S-NO-HSA-hHTX vs. 12 h-control-hHTX: *p* = 0.0008; normalized expression to ACTB).

Grafts that were subjected to ischemia but not transplanted showed no significant difference in GATA2 expression levels compared to control hearts without ischemia. In the non-transplanted groups, there was also no significant difference in GATA2-expression depending on whether donors received S-NO-HSA (−5.1 ± 0.33 vs. −5.74 ± 0.22; *p* = 0.134; 12 h-SNO-HSA-no-hHTX vs. 12 h-control-no-hHTX; normalized expression to ACTB).

A positive correlation was found between GATA2 and miR-126-3p expression levels of all samples [*r* = 0.496, *p* = 0.0006; Supplemental Figure S1 (Supplementary Appendix S1)].

### Role of Nitric Oxide on the Expression of Markers for Fibrosis in Human Cardiac Fibroblasts

**FIGURE 6:**
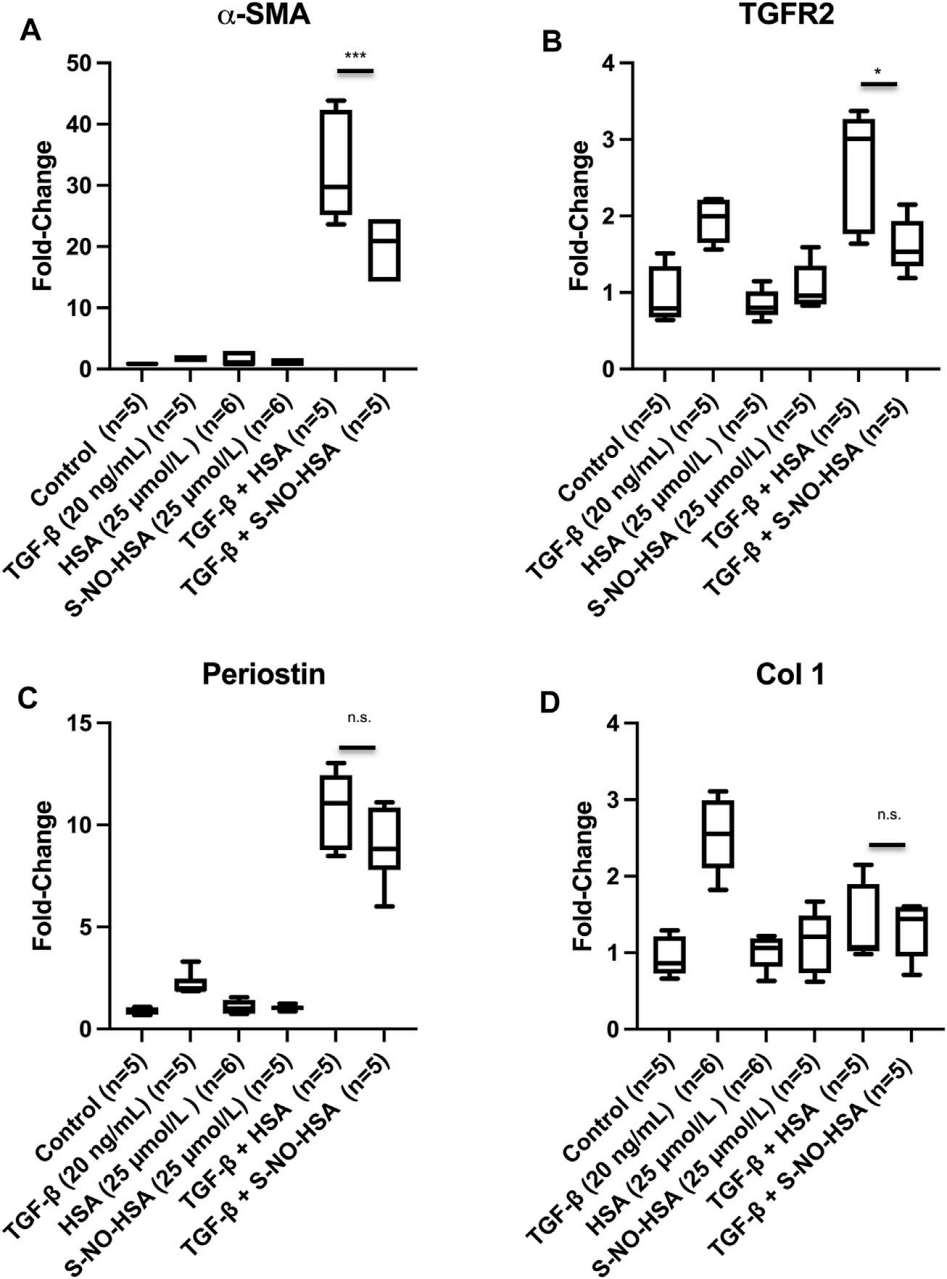
Expression levels of *α*-smooth muscle actin [αSMA, panel **(A)**], transforming growth factor-β type II receptors [TGFBR2, panel **(B)**], perostin **(C)** and collagen I **(D)** in transforming growth factor (TGF-β) stimulated human ventricular cardiac fibroblasts. Cells were treated for 24 h as follows: No treatment—control; TGFβ (20 ng/ml); HSA (25 μmol/L); S-NO-HSA (25 μmol/L); TGF-β (20 ng/ml) + HSA (25 μmol/L) and TGFβ (20 ng/ml) + S-NO-HSA (25 μmol/L). Total RNA was extracted, and expression of target genes (Supplementary Table S5) were assessed by RT-qPCR. mean ± SD (*n* = 6 per treatment); ***p < 0.001; *p < 0.05; n.s. = non-significant.

To further evaluate the role of an intact eNOS (endothelium) and its effect on fibrosis we utilized S-NO-HSA as a tool. S-NO-HSA at a concentration of 25 μmol/L releases NO in a physiological range of approximately 150 nmol/L in cell culture medium or physiological saline (20). The potential anti-fibrotic effect of intact eNOS (intact endothelium) was studied in human cardiac fibroblasts, which were cultivated and treated with TGF-β in order to stimulate fibroblast to myofibroblast transition. As appropriate control to 25 μmol/L S-NO-HSA 25 μmol/L HSA was used. In direct comparison NO released *via* S-NO-HSA significantly decreased α-SMA mRNA levels (Figure 6A, panel a; *p* = 0.0006) and transforming growth factor-β (TGF-β) type II receptors (TGFBR2) expression levels (Figure 6B; *p* = 0.0139) in TGF-β stimulated fibroblasts. In addition, perostin levels (another marker of activated fibroblast) was reduced with S-NO- HSA but did not reach significance compared to HSA (Figure 6C). Both HSA and S-NO-HSA reduced collagen I expression levels in TGF-β stimulated fibroblasts (revealing no specific NO effect; Figure 6D).

However, it has to be mentioned that in TGF-β stimulated fibroblasts both HSA and S-NO-HSA further increased α SMA levels (HSA: 18-fold) and perostin expression levels (HSA: 4.9-fold) in TGF-β stimulated fibroblasts. It is known that HSA can enhance mRNA expression levels as we observed in these two cases (21).

## Discussion

Optimizing preservation methods is crucial, as improving cold storage enables increasing the donor pool by long-distance procurements and acceptance of marginal donors. Previous studies have demonstrated the superior cardioprotective effect of HTK-N (22), an effect that can even be augmented by the addition of the nitric oxide donor S-NO-HSA (8). However, these studies have focused on acute functional parameters, and little is known about long-term effects on the myocardium after transplantation.

In the present study, donor pre-treatment with intravenous S-NO-HSA prior to graft procurement significantly reduced the long-term development of interstitial fibrosis in heterotopically transplanted murine cardiac isografts. This effect was accompanied by preservation of myocardial GATA2 and miR-126-3p expression. Whilst depletion of miR-126-3p was present in all grafts subjected to cold ischemia, this effect was significantly attenuated by donor pre-treatment with S-NO-HSA.

Our data suggests that miR-126-3p downregulation seems to be related to the ischemic period per se, since downregulation was also observed in non-transplanted grafts after the ischemic period. MiR-126-3p downregulation seems also to be less dependent on the duration of ischemic period, since no significant difference in miR-126-3p expression between transplanted grafts after 1 h and 12 h of ischemia was observed.

In contrast to miR-126-3p levels, GATA2 expression was markedly reduced only in transplanted grafts, and this effect was reversed in the S-NO-HSA group, suggesting preserved endothelial cell function. Preservation of myocardial miR-126-3p levels by S-NO-HSA administration is an important novel finding and suggests that dysregualation of miR-126-3p in the myocardium is primarily caused by ischemia. In further consequence, depletion of miR-126-3p may play a causative role in the development of cardiac fibrosis.

Mechanistically, there is evidence that miR-126-3p has pro-angiogenic properties by degradation of negative regulators in the vascular endothelial growth factor pathway, phosphoinositol-3 kinase regulatory subunit 2 (PI3KR2) and sprouty related protein 1 (SPRED1), thereby maintaining the integrity of blood vessels (23). In line with our results, previous studies demonstrated that depletion of miR-126-3p is associated with impaired cardiac and vascular function (14). Yang et al. found that overexpression of miR-126-3p protected human cardiac microvascular endothelial cells against hypoxia/reoxygenation injury via a mechanism activating the PI3K/Akt/eNOS signaling pathway (24). Accordingly, we found that cold ischemia is accompanied by a marked decline of miR-126-3p in transplanted hearts. Furthermore, it has been shown that miR-126-3p does not only affect endothelial cells, but also initiates cardioprotection against ischemia-reperfusion-injury in cardiomyocytes (25).

Dysregulated circulating miRs are potential biomarkers for cardiovascular diseases: (13, 26). A recent clinical study has shown that *circulating* miR-126-3p was upregulated in patients with CAV compared to transplanted patients without CAV (13). In contrast, downregulation of *tissue* miR-126-3p has been described in a very recent study in myocardial biopsies of transplant recipients with allograft vasculopathy, which is in line with our findings (14). Nevertheless, further preclinical and clinical studies are warranted to clarify the role and spatial-temporal expression pattern of miR-126-3p in HTX.

A recent pioneering study by Hartmann et al. demonstrated that GATA2 regulates miR-126-3p in endothelial cells (12). In line with this finding, we found a correlation between mir-126-3p expression and GATA2 levels. In addition, we observed a decline of GATA2 expression in transplanted grafts. These effects were partially counteracted by donor pretreatment with S-NO-HSA. In our study, we did not investigate the mode of action how S-NO-HSA modifies GATA2, however it is tempting to speculate that preserved endothelial cell viability may lead to maintainance of GATA2 levels and subsequent functional improvement.

An ischemia duration-dependent increase in myocardial fibrosis in transplanted cardiac isografts 60 days after transplantation was detected. The extent of fibrosis was significantly attenuated when donors were pretreated with S-NO-HSA before procurement.

NO deficiency due to its consumption by superoxide (O_2_^−^), produced in high concentrations during ischemia and reperfusion are known to play an important role in the pathophysiology of I/R injury (15). The mechanism by which S-NO-HSA as an exogenous NO-donor can protect the dysfunction of the endothelium and prevent excessive O_2_^−^ formation is based on prevention of eNOS uncoupling (10, 15). The uncoupled eNOS can intermittently produce both NO and superoxide (27, 28). It is of note that the slow and long-lasting release of NO by S-NO-HSA compared to small molecular weight S-nitroso thiols is a special feature of the applied drug. Mean arterial blood pressure is not affected at a dose of 0.1 μmol/kg/h of S-NO-HSA (29). Recently, this difference in kinetics of NO release by S-NO-HSA has also been demonstrated intracellularly by live-cell imaging of nitric oxide dynamics with novel FP-based probes (20).

In the present study, administration of S-NO-HSA as pretreatment to the donor may increase/preserve NO bioavailibility due to prevention of eNOS uncoupling during the cold ischemia and reperfusion period, leading to a decrease in oxidative/nitroxidative stress induced by O_2_^−^ and peroxynitrite (ONOO^−^) formation, and maintainance of endothalial cell integrity and function during the period of I/R (10). In the pretreatment phase, NO provided by S-NO-HSA may downregulate eNOS activity through feedback inhibition and thereby preserve its function (prevent eNOS uncoupling) (30). Therefore, in this setting, beneficial effects can be explained by two mechanisms: on the one hand, pretreatment with S-NO-HSA downregulates eNOS prior to ischemia and reperfusion by supplementing NO and thereby preserving and stabilizing its function during the prolonged ischemic phase, leading to sufficient NO production after transplantation (reperfusion), on the other hand, the improved NO production has further an indirect/direct positive inotropic and lusitropic effect on the myocardial cell and thereby preserves cardiac function (10). S-NO-HSA administration for only 20 min prior to donor heart procurement seems to minimize/prevent eNOS uncoupling during the 12 h storage in HTK-N solution at 4°C and subsequent transplantation. Interestingly, this pretreatment with the NO-donor is sufficient to reduce fibrosis after 60 days of transplantation. NO has also been reported to act as an antifibrotic effector in animal models of experimental fibrosis and a loss of NO bioavailability in eNOS knock-out mice resulted in increased fibrosis (6, 31, 32). These data are in line with our observed results and emphasize the importance of NO bioavailability in the prevention of fibrosis (32).

As S-NO-HSA prevents eNOS uncoupling in our transplant model and in further consequence reduces fibrosis, we utilized S-NO-HSA as NO donor to simulate a preserved endothelium in experiments with human fibroblasts and TGF-ß stimulation. NO provided by S-NO-HSA at physiologically relevant concentration significantly decreased *α*-smooth muscle actin (*α*-SMA) levels as well as TGFRII expression levels in TGF-β stimulated fibroblasts when compared to HSA (Figures 6A,B). Park et al. have recently demonstrated that NO (*via* nitrite) significantly decreased α-SMA expression in TGF-ß stimulated fibroblasts thereby attenuating myofibroblast differentiation of human keratocytes (33). In addition, inhibiting the production of NO causes endothelial cells to produce factors that promote the expression in fibroblasts of α-SMA and collagen type I (34). In our model we could not attribute the reduction of expression of collagen type I to NO as both HSA and S-NO-HSA showed a reduction (Figure 6D). It has also been shown that increased levels of TGFRII from matrix-producing interstitial cells such as fibroblast are sufficient to increase the severity of fibrosis (35). The expression levels of periostin (a marker of activated fibroblasts) (36) was also reduced with S-NO-HSA compared to HSA but did not reach a level of significance (Figure 6C). Taking together the data reveals the importance of NO (and intact eNOS) in the prevention of fibrosis. The data with the NO donor S-NO-HSA on expression of markers for fibrosis in TGF-β stimulated human cardiac fibroblasts are in line with our finding that hearts transplanted after prolonged cold ischemia showed significantly reduced fibrosis 60 days after transplantation when donors were pretreated with S-NO-HSA.

### Limitations

The animal model used in the present study is not able to fully mimic the clinical scenario of cardiac transplantation. After heterotopic transplantation, the graft is perfused and beating, but the left ventricle is unloaded, leading to graft atrophy and thrombus formation in the left ventricular cavity over time. However, quantification of fibrosis is possible in the right ventricular myocardium and the interventricular septum.

Our study did not aim to investigate the influence of immunologic responses and we therefore did not choose an allograft model. In the isogenic transplantation model applied, we did not expect nor observe graft vasculopathy.

However, the control role of miR-126-3p on the progression of neointima formation and vascular smooth muscle cell proliferation has been demonstrated in previous studies (37), suggesting that the depletion of miR-126-3p in transplanted hearts demonstrated in our study may be an indicator for CAI. This let us hypothesize that preservation of miR-126-expression may also inhibit CAV pathogenesis. Further studies are also required to assess other variables with, e.g., PET-MRI which will enable to establish correlations with graft viability and functional analysis of the transplanted hearts.

## Conclusion

Intravenous administration of S-NO-HSA to the donor prior to organ procurement significantly attenuated myocardial interstitial fibrosis, and lead to preservation of both GATA2 and miR-126-3p expression in cardiac isografts. These results indicate that the signaling pathways involving GATA2 and miR-126-3p participate in the pathogenesis of CAI, and targeting miR-126-3p might represent a potential novel therapeutic approach to limit ischaemia-mediated cardiac and vascular dysfunction in heart transplant recipients. S-NO-HSA may represent a useful therapeutic adjunct to pre-transplant graft preservation, which is clinically easily applicable without requiring a direct intervention on the organ recipient.

## Data Availability

The raw data supporting the conclusion of this article will be made available by the authors, without undue reservation.

## Abbreviations

CAI, Chronic allograft injury; CAV, cardiac allograft vasculopathy; eNOS, endothelial nitric oxide synthase; HTK-N, histidine-tryptophan-α-ketoglutarate-N; hHTX, heterotopic heart transplantation; HTX, heart transplantation; I/R, ischemia/reperfusion; IVC, inferior vena cava; miR, microRNA; NO, nitric oxide; RT-qPCR, real time quantitative polymerase chain reaction; S-NO-HSA, S-nitroso-human-serum-albumin.

## References

[1] Yusen RD Edwards LB Kucheryavaya AY Benden C Dipchand AI Goldfarb SB The Registry of the International Society for Heart and Lung Transplantation: Thirty-Second Official Adult Lung and Heart-Lung Transplantation Report-2015; Focus Theme: Early Graft Failure. J Heart Lung Transplant (2015) 34(10):1264–77. 10.1016/j.healun.2015.08.014 26454740

[2] Davis SF Yeung AC Meredith IT Charbonneau F Ganz P Selwyn AP Early Endothelial Dysfunction Predicts the Development of Transplant Coronary Artery Disease at 1 Year Posttransplant. Circulation (1996) 93(3):457–62. 10.1161/01.cir.93.3.457 8565162

[3] Lee JH Okada K Khush K Kobayashi Y Sinha S Luikart H Coronary Endothelial Dysfunction and the Index of Microcirculatory Resistance as a Marker of Subsequent Development of Cardiac Allograft Vasculopathy. Circulation (2017) 135(11):1093–5. 10.1161/circulationaha.116.025268 28289008 PMC5354083

[4] Bedi DS Riella LV Tullius SG Chandraker A. Animal Models of Chronic Allograft Injury: Contributions and Limitations to Understanding the Mechanism of Long-Term Graft Dysfunction. Transplantation (2010) 90(9):935–44. 10.1097/tp.0b013e3181efcfbc 20703180

[5] Wilck N Markó L Balogh A Kräker K Herse F Bartolomaeus H Nitric Oxide-Sensitive Guanylyl Cyclase Stimulation Improves Experimental Heart Failure with Preserved Ejection Fraction. JCI Insight (2018) 3(4):e96006. 10.1172/jci.insight.96006 PMC5916255 29467337

[6] Chung MP Monick MM Hamzeh NY Butler NS Powers LS Hunninghake GW. Role of Repeated Lung Injury and Genetic Background in Bleomycin-Induced Fibrosis. Am J Respir Cell Mol Biol (2003) 29(3 Pt 1):375–80. 10.1165/rcmb.2003-0029OC 12676806

[7] Semsroth S Fellner B Trescher K Bernecker OY Kalinowski L Gasser H S-nitroso Human Serum Albumin Attenuates Ischemia/reperfusion Injury after Cardioplegic Arrest in Isolated Rabbit Hearts. J Heart Lung Transplant (2005) 24(12):2226–34. 10.1016/j.healun.2005.08.004 16364875

[8] Trescher K Dzilic E Kreibich M Gasser H Aumayr K Kerjaschki D The Nitric Oxide Donor, S-Nitroso Human Serum Albumin, as an Adjunct to HTK-N Cardioplegia Improves protection during Cardioplegic Arrest after Myocardial Infarction in Rats. Interact Cardiovasc Thorac Surg (2015) 20(3):387–94. 10.1093/icvts/ivu383 25468794

[9] Griscavage JM Hobbs AJ Ignarro LJ. Negative Modulation of Nitric Oxide Synthase by Nitric Oxide and Nitroso Compounds. Adv Pharmacol (1995) 34:215–34. 10.1016/s1054-3589(08)61088-1 8562436

[10] Hallström S Franz M Gasser H Vodrazka M Semsroth S Losert UM S-nitroso Human Serum Albumin Reduces Ischaemia/reperfusion Injury in the Pig Heart after Unprotected Warm Ischaemia. Cardiovasc Res (2008) 77(3):506–14. 10.1093/cvr/cvm052 18006447

[11] De Val S Black BL. Transcriptional Control of Endothelial Cell Development. Developmental Cell (2009) 16(2):180–95. 10.1016/j.devcel.2009.01.014 19217421 PMC2728550

[12] Hartmann D Fiedler J Sonnenschein K Just A Pfanne A Zimmer K MicroRNA-Based Therapy of GATA2-Deficient Vascular Disease. Circulation (2016) 134(24):1973–90. 10.1161/circulationaha.116.022478 27780851

[13] Singh N Heggermont W Fieuws S Vanhaecke J Van Cleemput J De Geest B. Endothelium-enriched microRNAs as Diagnostic Biomarkers for Cardiac Allograft Vasculopathy. J Heart Lung Transplant (2015) 34(11):1376–84. 10.1016/j.healun.2015.06.008 26198441

[14] Heggermont WA Delrue L Dierickx K Dierckx R Verstreken S Goethals M Low MicroRNA-126 Levels in Right Ventricular Endomyocardial Biopsies Coincide with Cardiac Allograft Vasculopathy in Heart Transplant Patients. Transpl Direct (2020) 6(5):e549. 10.1097/txd.0000000000000995 PMC7213604 32548243

[15] Hallström S Gasser H Neumayer C Fügl A Nanobashvili J Jakubowski A S-nitroso Human Serum Albumin Treatment Reduces Ischemia/reperfusion Injury in Skeletal Muscle via Nitric Oxide Release. Circulation (2002) 105(25):3032–8. 10.1161/01.cir.0000018745.11739.9b 12081999

[16] Hasegawa T Visovatti SH Hyman MC Hayasaki T Pinsky DJ. Heterotopic Vascularized Murine Cardiac Transplantation to Study Graft Arteriopathy. Nat Protoc (2007) 2(3):471–80. 10.1038/nprot.2007.48 17406609

[17] Liu F Kang SM. Heterotopic Heart Transplantation in Mice. JoVE (2007) 6:238. 10.3791/238 PMC2557111 18997886

[18] Carpenter AE Jones TR Lamprecht MR Clarke C Kang I Friman O CellProfiler: Image Analysis Software for Identifying and Quantifying Cell Phenotypes. Genome Biol (2006) 7(10):R100. 10.1186/gb-2006-7-10-r100 17076895 PMC1794559

[19] Perera-Gonzalez M Kiss A Kaiser P Holzweber M Nagel F Watzinger S The Role of Tenascin C in Cardiac Reverse Remodeling Following Banding-Debanding of the Ascending Aorta. Int J Mol Sci (2021) 22(4):2023. 10.3390/ijms22042023 33670747 PMC7921966

[20] Eroglu E Rost R Bischof H Blass S Schreilechner A Gottschalk B Application of Genetically Encoded Fluorescent Nitric Oxide (NO•) Probes, the geNOps, for Real-Time Imaging of NO• Signals in Single Cells. J Vis Exp (2017) 121. 10.3791/55486 PMC5408997 28362417

[21] Higai K Satake M Nishioka H Azuma Y Matsumoto K. Glycated Human Serum Albumin Enhances Macrophage Inflammatory Protein-1β mRNA Expression through Protein Kinase C-δ and NADPH Oxidase in Macrophage-like Differentiated U937 Cells. Biochim Biophys Acta (Bba) - Gen Subjects (2008) 1780(2):307–14. 10.1016/j.bbagen.2007.11.010 18155175

[22] Trescher K Hasun M Baumgartner A Dietl W Wolfsberger M Hallström S New HTK-N46b Cardioplegia Provides superior protection during Ischemia/reperfusion in Failing Hearts. J Cardiovasc Surg (Torino) (2013) 54(3):413–21. 23389583

[23] Fish JE Santoro MM Morton SU Yu S Yeh R-F Wythe JD miR-126 Regulates Angiogenic Signaling and Vascular Integrity. Developmental Cell (2008) 15(2):272–84. 10.1016/j.devcel.2008.07.008 18694566 PMC2604134

[24] Yang H-H Chen Y Gao C-Y Cui Z-T Yao J-M. Protective Effects of MicroRNA-126 on Human Cardiac Microvascular Endothelial Cells against Hypoxia/Reoxygenation-Induced Injury and Inflammatory Response by Activating PI3K/Akt/eNOS Signaling Pathway. Cell Physiol Biochem (2017) 42(2):506–18. 10.1159/000477597 28578351

[25] Shi H Chen L Wang H Zhu S Dong C Webster KA Synergistic Induction of miR-126 by Hypoxia and HDAC Inhibitors in Cardiac Myocytes. Biochem Biophysical Res Commun (2013) 430(2):827–32. 10.1016/j.bbrc.2012.11.061 PMC3638006 23201405

[26] Neumann A Napp LC Kleeberger JA Benecke N Pfanne A Haverich A MicroRNA 628-5p as a Novel Biomarker for Cardiac Allograft Vasculopathy. Transplantation (2017) 101(1):e26–e33. 10.1097/tp.0000000000001477 27653298

[27] Huk I Nanobashvili J Neumayer C Punz A Mueller M Afkhampour K l -Arginine Treatment Alters the Kinetics of Nitric Oxide and Superoxide Release and Reduces Ischemia/Reperfusion Injury in Skeletal Muscle. Circulation (1997) 96(2):667–75. 10.1161/01.cir.96.2.667 9244241

[28] Pou S Pou WS Bredt DS Snyder SH Rosen GM. Generation of Superoxide by Purified Brain Nitric Oxide Synthase. J Biol Chem (1992) 267(34):24173–6. 10.1016/s0021-9258(18)35745-4 1280257

[29] Jakubowski A Maksimovich N Olszanecki R Gebska A Gasser H Podesser BK S-nitroso Human Serum Albumin Given after LPS challenge Reduces Acute Lung Injury and Prolongs Survival in a Rat Model of Endotoxemia. Naunyn-schmied Arch Pharmacol (2009) 379(3):281–90. 10.1007/s00210-008-0351-2 18854985

[30] Ignarro LJ Napoli C. Novel Features of Nitric Oxide, Endothelial Nitric Oxide Synthase, and Atherosclerosis. Curr Diab Rep (2005) 5(1):17–23. 10.1007/s11892-005-0062-8 15663912

[31] Yoshimura S Nishimura Y Nishiuma T Yamashita T Kobayashi K Yokoyama M. Overexpression of Nitric Oxide Synthase by the Endothelium Attenuates Bleomycin-Induced Lung Fibrosis and Impairs MMP-9/TIMP-1 Balance. Respirology (2006) 11(5):546–56. 10.1111/j.1440-1843.2006.00894.x 16916326

[32] Dooley A Bruckdorfer KR Abraham DJ. Modulation of Fibrosis in Systemic Sclerosis by Nitric Oxide and Antioxidants. Cardiol Res Pract (2012) 2012:521958. 10.1155/2012/521958 22111028 PMC3206384

[33] Park J-H Kim M Yim B Park CY. Nitric Oxide Attenuated Transforming Growth Factor-β Induced Myofibroblast Differentiation of Human Keratocytes. Sci Rep (2021) 11(1):8183. 10.1038/s41598-021-87791-x 33854158 PMC8046755

[34] Sun YBY Qu X Li X Nikolic-Paterson DJ Li J. Endothelial Dysfunction Exacerbates Renal Interstitial Fibrosis through Enhancing Fibroblast Smad3 Linker Phosphorylation in the Mouse Obstructed Kidney. PLoS One (2013) 8(12):e84063. 10.1371/journal.pone.0084063 24391884 PMC3877161

[35] Khalil H Kanisicak O Prasad V Correll RN Fu X Schips T Fibroblast-specific TGF-β-Smad2/3 Signaling Underlies Cardiac Fibrosis. J Clin Invest (2017) 127(10):3770–83. 10.1172/jci94753 28891814 PMC5617658

[36] Kashima TG Nishiyama T Shimazu K Shimazaki M Kii I Grigoriadis AE Periostin, a Novel Marker of Intramembranous Ossification, Is Expressed in Fibrous Dysplasia and in C-Fos-Overexpressing Bone Lesions. Hum Pathol (2009) 40(2):226–37. 10.1016/j.humpath.2008.07.008 18799196

[37] Jansen F Stumpf T Proebsting S Franklin BS Wenzel D Pfeifer P Intercellular Transfer of miR-126-3p by Endothelial Microparticles Reduces Vascular Smooth Muscle Cell Proliferation and Limits Neointima Formation by Inhibiting LRP6. J Mol Cell Cardiol (2017) 104:43–52. 10.1016/j.yjmcc.2016.12.005 28143713

