# Supplementary material for: Single Donor Infusion of S-Nitroso-Human-Serum-Albumin Attenuates Cardiac Isograft Fibrosis and Preserves Myocardial Micro-RNA-126-3p in a Murine Heterotopic Heart Transplant Model
Source: Transpl Int. 2022 Apr 13;35:10057. doi: 10.3389/ti.2022.10057 (PMC9045410; doi:10.3389/ti.2022.10057)
Supplement: Supplementary file 1 [file DataSheet1.docx]

Single donor infusion of S-Nitroso-Human-Serum-Albumin attenuates cardiac isograft fibrosis and preserves myocardial Micro-RNA-126-3p in a murine heterotopic heart transplant model

Anne-Kristin Schaefer MD, Attila Kiss PhD, André Oszwald MD, et.al.

**Table of contents**

1. **S-NO-HSA preparation**
2. **Donor heart procurement**
3. **Heterotopic abdominal heart transplantation**
4. **Myocardial and blood sample collection**
5. **Assessment of miR-126 and GATA2 expression**
6. **Histological analysis**
7. **Supplementary tables and figures**
8. **S-NO-HSA preparation**

HSA was processed to yield a maximal free thiol group at position Cys-34 (SH>0.8 mol/mol protein). Intermolecular disulfides (mixed disulfides) were disassembled prior to nitrosation. The starting material (20 % HSA; Biotest) was reduced by mercaptoethanol (10 to 20-fold molar excess; buffer [mmol/L]: sodium phosphate 1, ethylenediaminetetraacetic acid 2, and sodium chloride 150 adjusted to pH=6.0-6.2 with hydrochloric acid (HCl); 12 to 48 hours at 4°C under nitrogen) and purified by means of gel-permeation chromatography (TSK-HW40F; mobile phase: H2O).

Thiol nitrosation was affected with sodium nitrite at a ratio of 1:1 to 1:1.5 of freely available thiol groups to nitrite in 0.2 mol/L HCl (pH=1.5-2.5) for 30 min at 25°C. After neutralization with 1 mol/L sodium hydroxide, S-NO-HSA was purified by gel-permeation chromatography (TSK-HW40F; mobile phase: H_2_O) and lyophilized. S-NO-HSA was dissolved in 0.9% saline solution.

1. **Donor heart procurement**

Donor mice were anesthetized by intraperitoneal injection of the mixture of xylazine (5 mg/kg) and ketamine (100 mg/kg), followed by catheterization of the femoral vein and intravenous infusion of S-NO-HSA (0.1 µmol/kg/h) dissolved in 0.9% saline solution, or 0.9% saline solution only (control groups) for 20 min, followed by thoracotomy and administration of 1 ml of HTK-N solution (4°C, Dr. Franz Köhler Chemie GmbH, Bensheim, Germany) supplemented with 100 IE of heparin via the inferior vena cava to arrest the heart. The ascending aorta and pulmonary trunk were divided. After ligation of the superior venae cavae, and en block ligation of the pulmonary veins, the graft was excised, flushed with heparinized HTK-N solution and stored in HTK-N solution at 4°C for either 1h or 12h.

1. **Heterotopic abdominal heart transplantation**

Analgesia was provided by subcutaneous injection of buprenorphine (0.1mg/kg bodyweight) and anaesthesia maintained with inhaled isoflurane. Briefly, after laparotomy and dissection of the infrarenal aorta and IVC, the abdominal aorta and IVC were cross-clamped infrarenally and directly proximal to the iliac bifurcation. After longitudinal aortotomy and venotomy, the donor’s acending aorta was anastomosed to the recipient’s abdominal aorta and the donor’s pulmonary trunk to the recipient’s IVC using running 10-0 nylon sutures. The duration of warm ischemia during the implantation process was standardized to 30 min.

1. **Myocardial and blood sample collection**

Sixty days after transplantation, recipient mice were anaesthetized with the mixture of ketamine and xylazine (0.1 ml/10 g bodyweight), and anaesthesia was confirmed by hind foot and tail pinch. Intravenous blood samples taken from the inferior vena cava were collected in EDTA tubes and immediately centrifuged. The obtained plasma was immediately stored at -80°C. The transplanted heart was excised and transversally cut at mid-papillary level. The base was immediately frozen at -80°C, and the apex fixed in 7.5% formaldehyde for histopathology analysis.

1. **Assessment of** **miR-126 and GATA2 expression**

RNA from myocardial tissue was isolated using the miRNeasy Mini Kit (Qiagen, Hilden, Germany) according to the manufacturers’ description. For miRNA analysis 10 ng RNA were reversed transcribed using the Universal cDNA Synthesis Kit II (Exiqon, Vedbaek, Denmark). For qPCR 4µL 1:40 diluted cDNA was mixed with 5 µL Exilent SYBR Green master mix (Exiqon, Vedbaek, Denmark) and 1µL LNA-enhanced miRNA primer (Exiqon, Vedbaek, Denmark) for U6 snRNA or miR-126-3p. For reverse transcription of mRNA the GrandScript cDNA synthesis kit (TATAA, Goteborg, Sweden) was used with an RNA input of 500ng. For qPCR 1:2 diluted cDNA was mixed with 5µL SYBR GrandMaster Mix (TATAA, Goteborg, Sweden), 2.2µL nuclease-free water and 0.8µL Primer (10µM) for ß-actin and GATA2. PCR amplification was performed in a Roche LC480 II instrument (Roche, Germany) and the cycle of quantification (Cq-values) was calculated with the second derivative method. Normalization was performed with the U6 snRNA for miRNA and ß-actin for mRNA analysis with following formula: normalized expression=reference-GOI. The following primer sequences were used:

GATA2: forward/reverse: GCAGAGAAGCAAGGCTCGC/CAGTTGACACACTCCCGGC;

Beta actin forward/reverse: GTCGAGTCGCGTCCACC/ GTCATCCATGGCGAACTGGT

1. **Histological analysis**

For quantification of interstitial fibrosis, sections of 4 µm were cut and stained with Sirius red. Images were acquired using an upright microscope and a CCD-camera (Axio Imager.M2 and Axiocam 512 color, Carl Zeiss, Aalen, Germany). At least two regions of myocardium each of the interventricular septum (IVS) and the right ventricle (RV) were selected for quantification. The areas of total and positively stained tissue within a region were quantified using CellProfiler. (17)

1. **Supplementary tables and figures**

**Figure S1:** Correlation of GATA2 and miR-126-3p expression levels in all analysed myocardial samples (n=45; thereof 19 samples without ischemia)

| **Groups** | **Donor**  **BW HTX (g)** | **Recipient**    **BW HTX (g)** | **Recipient**  **BW sacrifice (g)** | **HW native**  **sacrifice (g)** | **HW Isograft**  **sacrifice (g)** | **Tibia length**    **(mm)** |
| --- | --- | --- | --- | --- | --- | --- |
| **12h-S-NO-HSA-hHTX**  **(n = 7)** | **25.1±1.32** | **24.8±1.29** | **31.2±1.54** | **0.15±0.03** | **0.10±0.08** | **19.08±0.43** |
| **1h-Control-hHTX**  **(n =5)** | **24.2±1.94** | **25.9±1.32** | **30.1±1.71** | **0.15±0.01** | **0.10±0.01** | **18.92±0.54** |
| **12h-Control-hHTX**  **(n =6)** | **24.6±1.65** | **25.1±1.97** | **29.8±1.71** | **0.16±0,013** | **0.11±0.04** | **18.90±0.41** |

**Table S2:** Baseline characteristics of experimental animals showed no significant difference between groups in donor and recipient body weight (BW) at the time of HTX and time of sacrifice, and heart weight (HW) and tibia length at the time of sacrifice 60 days after HTX. BW=bodyweight in grams (g), HW=heart weight in grams (g).

| **Group comparison** | **Mean difference** | **95% CI of significant diff.** | **Adjusted p-value** |
| --- | --- | --- | --- |
| **no ischemia vs. 12h-control-hHTX** | **1,406** | **0,9485 to 1,863** | **<0,0001** |
| **no ischemia vs. 12h-SNO-hHTX** | **0,7369** | **0,2463 to 1,228** | **0,0008** |
| **no ischemia vs. 1h-control-hHTX** | **1,359** | **0,8683 to 1,850** | **<0,0001** |
| **no ischemia vs. 12h-control-no-hHTX** | **1,578** | **1,087 to 2,069** | **<0,0001** |
| **no ischemia vs. 12h-SNO-no-hHTX** | **0,9159** | **0,4253 to 1,407** | **<0,0001** |
| **12h-control-hHTX vs. 12h-SNO-hHTX** | **-0,6687** | **-1,260 to -0,07759** | **0,0187** |
| 12h-control-hHTX vs. 1h-control-hHTX | -0,04667 | -0,6377 to 0,5444 | 0,9999 |
| 12h-control-hHTX vs. 12h-control-no-hHTX | 0,1723 | -0,4187 to 0,7634 | 0,9506 |
| 12h-control-hHTX vs.12h-SNO-no-hHTX | -0,4897 | -1,081 to 0,1014 | 0,1545 |
| **12h-SNO-hHTX vs. 1h-control-hHTX** | **0,6220** | **0,004645 to 1,239** | **0,0474** |
| **12h-SNO-hHTX vs. 12h-control-no-hHTX** | **0,8410** | **0,2236 to 1,458** | **0,0028** |
| 12h-SNO-hHTX vs. 12h-SNO-no-hHTX | 0,1790 | -0,4384 to 0,7964 | 0,9517 |
| 1h-control vs. 12h-control-no-hHTX | 0,2190 | -0,4384 to 0,7964 | 0,8930 |
| 1h-control-hHTX vs. 12h-SNO-no-hHTX | -0,4430 | -1,060 to 0,1744 | 0,2841 |
| **noHTX 12h-control vs. noHTX 12h-SNO** | **-0,6620** | **-1,279 to -0,04465** | **0,0294** |

**Table S3:** One-way ANOVA of miR-126-3p, Tukey’s multiple comparisons test.

| **Group comparison** | **Mean difference** | **95% CI of significant diff.** | **Adjusted p-value** |
| --- | --- | --- | --- |
| **no ischemia vs. 12h-control-hHTX** | **1,822** | **1,249 to 2,395** | **<0,0001** |
| no ischemia vs. 12h-SNO-hHTX | 0,6335 | -0,03957 to 1,307 | 0,0752 |
| **no ischemia vs. 1h-control-hHTX** | **0,7077** | **0,09278 to 1,323** | **0,0161** |
| no ischemia vs. 12h-control-no-hHTX | 0,3767 | -0,2382 to 0,9917 | 0,4548 |
| no ischemia vs. 12h-SNO-no-hHTX | -0,2823 | -0,8972 to 0,3327 | 0,7401 |
| **12h-control-hHTX vs. 12h-SNO-hHTX** | **-1,189** | **-1,979 to -0,3990** | **0,0008** |
| **12h-control-hHTX vs. 1h-control-hHTX** | **-1,115** | **-1,855 to -0,3736** | **0,0008** |
| **12h-control-hHTX vs. 12h-control-no-hHTX** | **-1,446** | **-2,186 to -0,7046** | **<0,0001** |
| **12h-control-hHTX vs.12h-SNO-no-hHTX** | **-2,105** | **-2,845 to -1,364** | **<0,0001** |
| 12h-SNO-hHTX vs. 1h-control-hHTX | 0,07425 | -0,7465 to 0,8950 | 0,9998 |
| 12h-SNO-hHTX vs. 12h-control-no-hHTX | -0,2568 | -1,077 to 0,5640 | 0,9338 |
| **12h-SNO-hHTX vs. 12h-SNO-no-hHTX** | **-0,9158** | **1,736 to -0,09501** | **0,0212** |
| 1h-control vs. 12h-control-no-hHTX | -0,3310 | -1,105 to 0,4428 | 0,7921 |
| **1h-control-hHTX vs. 12h-SNO-no-hHTX** | **-0,9900** | **-1,764 to -0,2162** | **0,0056** |
| noHTX 12h-control vs. noHTX 12h-SNO | -0,6590 | -1,433 to 0,1148 | 0,1340 |

**Table S4:** One-way ANOVA of GATA2, Tukey’s multiple comparisons test.

| **Gene** | **Forward 5’-3’** | **Primer lenght (bp)** | **Reverse 5’-3’** | **Primer lenght (bp)** |
| --- | --- | --- | --- | --- |
| GAPDH (HKG) | GGAAGGAAATGAATGGGCAG | 20 | CCCAATACGACCAAATCAGAG | 21 |
| TGFBR2 | CCAACAACATCAACCACAAC | 20 | CCACTGTCTCAAACTGCTC | 19 |
| α-SMA | GTACCACCATGTACCCAGGC | 20 | GCTGGAAGGTAGACAGCGAA | 20 |
| Col I | AGTGGTTTGGATGGTGCCAA | 20 | GCACCATCATTTCCACGAGC | 20 |
| Periostin | ACACACCCGTGAGGAAGTTG | 20 | CACTGAGAACGACCTTCCCT | 20 |

**Table S5:** **List of primers sequences.** GAPDH: Glyceraldehyde 3-phosphate dehydrogenase (housekeeping gene), α-SMA: alpha smooth muscle actin, TGFBR2: TGF beta receptor 2; Col I: collagen I and periostin.
